# *N*′-[4-(2-Fur­yl)but-3-en-2-yl­idene]­iso­nicotino­hydrazide

**DOI:** 10.1107/S1600536808033242

**Published:** 2008-10-22

**Authors:** Zhi-Gang Yin, Yu-Zhen Chen, Heng-Yu Qian, Jie Hu

**Affiliations:** a Key Laboratory of Surface and Interface Science of Henan, School of Materials and Chemical Engineering, Zhengzhou University of Light Industry, Zhengzhou 450002, People’s Republic of China

## Abstract

The mol­ecule of the title Schiff base compound, C_14_H_13_N_3_O_2_, is not perfectly planar; the furyl and pyridine rings are twisted with respect to each other along the C_4_N_2_C_2_ organic chain, making a dihedral angle of 13.3 (1)°. The occurence of N—H⋯O hydrogen bonds builds up a chain developing parallel to the *c* axis.

## Related literature

For background, see: Kahwa *et al.* (1986 ▶); Santos *et al.* (2001 ▶).
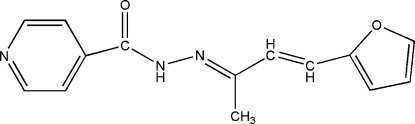


## Experimental

### 

#### Crystal data

C_14_H_13_N_3_O_2_
*M* *_r_* = 255.27
Monoclinic, 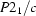

*a* = 16.6325 (14) Å
*b* = 9.3572 (8) Å
*c* = 8.3554 (7) Å
β = 100.912 (1)°
*V* = 1276.87 (19) Å^3^
*Z* = 4
Mo *K*α radiation
μ = 0.09 mm^−1^
*T* = 293 (2) K
0.25 × 0.23 × 0.16 mm

#### Data collection

Bruker SMART CCD area-detector diffractometer
Absorption correction: multi-scan (*SADABS*; Bruker, 1998 ▶) *T*_min_ = 0.965, *T*_max_ = 0.978
11728 measured reflections
3151 independent reflections
2124 reflections with *I* > 2σ(*I*)
*R*_int_ = 0.026

#### Refinement

*R*[*F* ^2^ > 2σ(*F* ^2^)] = 0.047
*wR*(*F* ^2^) = 0.132
*S* = 1.03
3151 reflections
173 parameters
H-atom parameters constrained
Δρ_max_ = 0.20 e Å^−3^
Δρ_min_ = −0.22 e Å^−3^

### 

Data collection: *SMART* (Bruker, 1998 ▶); cell refinement: *SAINT* (Bruker, 1998 ▶); data reduction: *SAINT*; program(s) used to solve structure: *SHELXS97* (Sheldrick, 2008 ▶); program(s) used to refine structure: *SHELXL97* (Sheldrick, 2008 ▶); molecular graphics: *ORTEPIII* (Burnett & Johnson, 1996 ▶), *ORTEP-3 for Windows* (Farrugia, 1997 ▶) and *PLATON* (Spek, 2003 ▶); software used to prepare material for publication: *SHELXL97*.

## Supplementary Material

Crystal structure: contains datablocks global, I. DOI: 10.1107/S1600536808033242/dn2389sup1.cif

Structure factors: contains datablocks I. DOI: 10.1107/S1600536808033242/dn2389Isup2.hkl

Additional supplementary materials:  crystallographic information; 3D view; checkCIF report

## Figures and Tables

**Table 1:** Hydrogen-bond geometry (Å, °)

*D*—H⋯*A*	*D*—H	H⋯*A*	*D*⋯*A*	*D*—H⋯*A*
N2—H2*A*⋯O2^i^	0.86	2.26	2.9289 (16)	134

Symmetry code: (i) 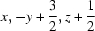.

**Fig. 1.**
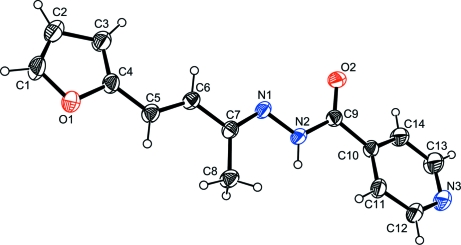
Molecular view of (I) with the atom-labeling scheme. Displacement ellipsoids are drawn at the 30% probability level. H atoms are represented as small spheres of arbitrary radii.

**Fig. 2.**
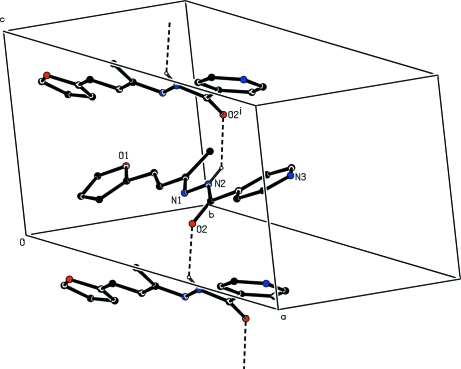
Partial packing view showing the N-H···O hydrogen bondings resulting in the formation of a chain parallel to the c axis. H atoms not involved in hydrogen bondings have been omitted for clarity. H bonds are shown as dashed lines. [Symmetry code: (i) x, -y+3/2, z+1/2]

## supplementary crystallographic information

### Comment

The chemistry of Schiff bases has attracted a great deal of interest in recent years. These compounds play an important role in the development of various proteins and enzymes(Kahwa *et al.*, 1986; Santos *et al.*, 2001). As part of our interest in the study of the coordination chemistry of Schiff bases, we have synthesized the title compound (I) and reported its cyrstal structure.

The molecule of the title compound is not perfectly planar, the furyl and the pyridine rings are twisted to each other along the C5/C6/C7/C8/N1/N2/C9 organic chain making a dihedral angle of 13.3 (1)°(Fig. 1). The organic chain is nearly planar with the largest deviation from the mean plane being 0.039 (1)Å at C7. The occurence of N-H···O hydrogen bonds builts up a chain developing parallel to the c axis (Fig. 2, Table 1).

### Experimental

Pyridine-4-carboxylic acid hydrazide (1 mmol, 0.137 g) was dissolved in anhydrous methanol, H_2_SO_4_ (98% 0.5 ml) was added to this, the mixture was stirred for several minitutes at 351 K, furylideneacetone (1 mmol 0.136 g) in methanol (8 ml) was added dropwise and the mixture was stirred at refluxing temperature for 2 h. The product was isolated and recrystallized in dichloromethane, brown single crystals of (I) was obtained after 5 d.

### Refinement

All H atoms were treated as riding on their parent atoms. Methyl H atoms were placed in calculated position with C—H=0.96Å and refined with *U*_iso_(H)=1.5*U*_eq_(C) Other H atoms were placed in calculated positions with C—H=0.93 Å, N—H=0.86Å and *U*_iso_(H)=1.2*U*_eq_(C,*N*).

### Crystal data

**Table:**

C_14_H_13_N_3_O_2_	*F*(000) = 536
*M**_r_* = 255.27	*D*_x_ = 1.328 Mg m^−^^3^
Monoclinic, *P*2_1_/*c*	Mo *K*α radiation, λ = 0.71073 Å
Hall symbol: -P 2ybc	Cell parameters from 2552 reflections
*a* = 16.6325 (14) Å	θ = 2.2–24.8°
*b* = 9.3572 (8) Å	µ = 0.09 mm^−^^1^
*c* = 8.3554 (7) Å	*T* = 293 K
β = 100.912 (1)°	Block, brown
*V* = 1276.87 (19) Å^3^	0.25 × 0.23 × 0.16 mm
*Z* = 4	

### Data collection

**Table:**

Bruker SMART CCD area-detector diffractometer	3151 independent reflections
Radiation source: fine-focus sealed tube	2124 reflections with *I* > 2σ(*I*)
graphite	*R*_int_ = 0.026
ω scans	θ_max_ = 28.3°, θ_min_ = 2.5°
Absorption correction: multi-scan (SADABS; Bruker, 1998)	*h* = −22→20
*T*_min_ = 0.965, *T*_max_ = 0.978	*k* = −12→12
11728 measured reflections	*l* = −11→11

### Refinement

**Table:**

Refinement on *F*^2^	Primary atom site location: structure-invariant direct methods
Least-squares matrix: full	Secondary atom site location: difference Fourier map
*R*[*F*^2^ > 2σ(*F*^2^)] = 0.047	Hydrogen site location: inferred from neighbouring sites
*wR*(*F*^2^) = 0.132	H-atom parameters constrained
*S* = 1.03	*w* = 1/[σ^2^(*F*_o_^2^) + (0.0611*P*)^2^ + 0.1613*P*] where *P* = (*F*_o_^2^ + 2*F*_c_^2^)/3
3151 reflections	(Δ/σ)_max_ = 0.005
173 parameters	Δρ_max_ = 0.20 e Å^−^^3^
0 restraints	Δρ_min_ = −0.21 e Å^−^^3^

### Special details

**Table:**

Geometry. All esds (except the esd in the dihedral angle between two l.s. planes) are estimated using the full covariance matrix. The cell esds are taken into account individually in the estimation of esds in distances, angles and torsion angles; correlations between esds in cell parameters are only used when they are defined by crystal symmetry. An approximate (isotropic) treatment of cell esds is used for estimating esds involving l.s. planes.
Refinement. Refinement of *F*^2^ against ALL reflections. The weighted *R*-factor *wR* and goodness of fit *S* are based on *F*^2^, conventional *R*-factors *R* are based on *F*, with *F* set to zero for negative *F*^2^. The threshold expression of *F*^2^ > σ(*F*^2^) is used only for calculating *R*-factors(gt) *etc*. and is not relevant to the choice of reflections for refinement. *R*-factors based on *F*^2^ are statistically about twice as large as those based on *F*, and *R*- factors based on ALL data will be even larger.

### Fractional atomic coordinates and isotropic or equivalent isotropic displacement parameters (Å^2^)

**Table:**

	*x*	*y*	*z*	*U*_iso_*/*U*_eq_	
C1	−0.34240 (10)	0.8729 (3)	0.0347 (2)	0.0765 (6)	
H1	−0.3937	0.9084	0.0428	0.092*	
C2	−0.33013 (10)	0.7542 (2)	−0.0421 (2)	0.0679 (5)	
H2	−0.3700	0.6929	−0.0970	0.081*	
C3	−0.24415 (10)	0.73836 (19)	−0.0245 (2)	0.0606 (4)	
H3	−0.2168	0.6641	−0.0654	0.073*	
C4	−0.20947 (9)	0.85094 (17)	0.06234 (19)	0.0510 (4)	
C5	−0.12679 (9)	0.89496 (16)	0.12619 (19)	0.0496 (4)	
H5	−0.1190	0.9739	0.1949	0.060*	
C6	−0.06062 (8)	0.82982 (14)	0.09315 (18)	0.0454 (3)	
H6	−0.0694	0.7556	0.0178	0.055*	
C7	0.02404 (8)	0.86343 (14)	0.16352 (17)	0.0424 (3)	
C8	0.04425 (9)	0.98435 (16)	0.2800 (2)	0.0547 (4)	
H8A	0.0616	0.9475	0.3883	0.082*	
H8B	−0.0033	1.0432	0.2765	0.082*	
H8C	0.0875	1.0403	0.2503	0.082*	
C9	0.21798 (8)	0.73017 (15)	0.13795 (17)	0.0439 (3)	
C10	0.30344 (8)	0.77406 (15)	0.21280 (17)	0.0450 (3)	
C11	0.32441 (9)	0.90777 (17)	0.2786 (2)	0.0540 (4)	
H11	0.2842	0.9759	0.2829	0.065*	
C12	0.40575 (10)	0.93870 (19)	0.3378 (2)	0.0653 (5)	
H12	0.4186	1.0289	0.3821	0.078*	
C13	0.44536 (11)	0.7198 (2)	0.2728 (3)	0.0838 (6)	
H13	0.4868	0.6536	0.2703	0.101*	
C14	0.36583 (10)	0.67795 (19)	0.2110 (2)	0.0651 (5)	
H14	0.3547	0.5865	0.1689	0.078*	
N1	0.07718 (7)	0.77927 (12)	0.11825 (15)	0.0466 (3)	
N2	0.15805 (7)	0.80590 (12)	0.18733 (15)	0.0475 (3)	
H2A	0.1701	0.8704	0.2613	0.057*	
N3	0.46678 (9)	0.84786 (19)	0.3355 (2)	0.0772 (5)	
O1	−0.26987 (7)	0.93681 (14)	0.10076 (15)	0.0705 (4)	
O2	0.20525 (6)	0.63417 (12)	0.03656 (13)	0.0573 (3)	

### Atomic displacement parameters (Å^2^)

**Table:**

	*U*^11^	*U*^22^	*U*^33^	*U*^12^	*U*^13^	*U*^23^
C1	0.0337 (9)	0.1172 (16)	0.0746 (13)	0.0063 (9)	0.0004 (8)	−0.0027 (12)
C2	0.0450 (10)	0.0840 (12)	0.0703 (12)	−0.0101 (9)	−0.0003 (8)	0.0038 (10)
C3	0.0477 (9)	0.0634 (9)	0.0681 (11)	−0.0004 (7)	0.0043 (8)	−0.0046 (8)
C4	0.0366 (8)	0.0627 (9)	0.0519 (9)	0.0068 (6)	0.0039 (6)	0.0025 (7)
C5	0.0401 (8)	0.0524 (8)	0.0533 (9)	0.0031 (6)	0.0012 (6)	−0.0001 (7)
C6	0.0390 (8)	0.0452 (7)	0.0489 (8)	0.0002 (6)	0.0002 (6)	0.0010 (6)
C7	0.0375 (7)	0.0415 (7)	0.0463 (8)	0.0010 (5)	0.0033 (6)	0.0039 (6)
C8	0.0425 (8)	0.0518 (8)	0.0649 (10)	0.0036 (6)	−0.0024 (7)	−0.0087 (7)
C9	0.0383 (7)	0.0502 (7)	0.0421 (8)	0.0003 (6)	0.0045 (6)	0.0005 (6)
C10	0.0357 (7)	0.0553 (8)	0.0434 (8)	−0.0004 (6)	0.0059 (6)	0.0005 (6)
C11	0.0382 (8)	0.0560 (9)	0.0693 (10)	−0.0045 (6)	0.0138 (7)	−0.0041 (8)
C12	0.0436 (9)	0.0708 (10)	0.0827 (12)	−0.0121 (8)	0.0147 (8)	−0.0144 (9)
C13	0.0405 (10)	0.0959 (14)	0.1109 (17)	0.0142 (9)	0.0039 (10)	−0.0275 (13)
C14	0.0449 (9)	0.0705 (10)	0.0769 (12)	0.0066 (8)	0.0039 (8)	−0.0174 (9)
N1	0.0329 (6)	0.0516 (6)	0.0526 (7)	−0.0027 (5)	0.0010 (5)	−0.0038 (5)
N2	0.0332 (6)	0.0528 (7)	0.0540 (7)	−0.0018 (5)	0.0021 (5)	−0.0111 (6)
N3	0.0382 (8)	0.0978 (12)	0.0931 (12)	−0.0041 (7)	0.0058 (7)	−0.0236 (9)
O1	0.0407 (6)	0.0913 (9)	0.0753 (8)	0.0138 (6)	0.0007 (5)	−0.0177 (7)
O2	0.0467 (6)	0.0676 (7)	0.0548 (7)	0.0016 (5)	0.0025 (5)	−0.0162 (5)

### Geometric parameters (Å, °)

**Table:**

C1—C2	1.317 (3)	C8—H8C	0.9600
C1—O1	1.366 (2)	C9—O2	1.2253 (17)
C1—H1	0.9300	C9—N2	1.3496 (18)
C2—C3	1.417 (2)	C9—C10	1.4980 (19)
C2—H2	0.9300	C10—C14	1.375 (2)
C3—C4	1.346 (2)	C10—C11	1.385 (2)
C3—H3	0.9300	C11—C12	1.380 (2)
C4—O1	1.3711 (18)	C11—H11	0.9300
C4—C5	1.438 (2)	C12—N3	1.327 (2)
C5—C6	1.332 (2)	C12—H12	0.9300
C5—H5	0.9300	C13—N3	1.330 (2)
C6—C7	1.4540 (19)	C13—C14	1.382 (2)
C6—H6	0.9300	C13—H13	0.9300
C7—N1	1.2926 (18)	C14—H14	0.9300
C7—C8	1.489 (2)	N1—N2	1.3822 (15)
C8—H8A	0.9600	N2—H2A	0.8600
C8—H8B	0.9600		
			
C2—C1—O1	111.08 (16)	H8B—C8—H8C	109.5
C2—C1—H1	124.5	O2—C9—N2	123.73 (13)
O1—C1—H1	124.5	O2—C9—C10	121.05 (13)
C1—C2—C3	106.49 (16)	N2—C9—C10	115.20 (12)
C1—C2—H2	126.8	C14—C10—C11	117.48 (14)
C3—C2—H2	126.8	C14—C10—C9	118.32 (14)
C4—C3—C2	107.16 (16)	C11—C10—C9	124.15 (13)
C4—C3—H3	126.4	C12—C11—C10	119.20 (15)
C2—C3—H3	126.4	C12—C11—H11	120.4
C3—C4—O1	109.08 (13)	C10—C11—H11	120.4
C3—C4—C5	134.95 (15)	N3—C12—C11	124.13 (16)
O1—C4—C5	115.91 (13)	N3—C12—H12	117.9
C6—C5—C4	124.18 (14)	C11—C12—H12	117.9
C6—C5—H5	117.9	N3—C13—C14	124.65 (17)
C4—C5—H5	117.9	N3—C13—H13	117.7
C5—C6—C7	126.31 (14)	C14—C13—H13	117.7
C5—C6—H6	116.8	C10—C14—C13	118.77 (16)
C7—C6—H6	116.8	C10—C14—H14	120.6
N1—C7—C6	114.42 (12)	C13—C14—H14	120.6
N1—C7—C8	124.89 (12)	C7—N1—N2	115.67 (11)
C6—C7—C8	120.68 (12)	C9—N2—N1	119.63 (12)
C7—C8—H8A	109.5	C9—N2—H2A	120.2
C7—C8—H8B	109.5	N1—N2—H2A	120.2
H8A—C8—H8B	109.5	C12—N3—C13	115.76 (15)
C7—C8—H8C	109.5	C1—O1—C4	106.18 (14)
H8A—C8—H8C	109.5		

### Hydrogen-bond geometry (Å, °)

**Table:**

*D*—H···*A*	*D*—H	H···*A*	*D*···*A*	*D*—H···*A*
N2—H2A···O2^i^	0.86	2.26	2.9289 (16)	134

Symmetry codes: (i) *x*, −*y*+3/2, *z*+1/2.
